# Multi-Optimization of Ultrasonic-Assisted Enzymatic Extraction of *Atratylodes macrocephala* Polysaccharides and Antioxidants Using Response Surface Methodology and Desirability Function Approach

**DOI:** 10.3390/molecules201219837

**Published:** 2015-12-11

**Authors:** Jin-Bao Pu, Bo-Hou Xia, Yi-Juan Hu, Hong-Jian Zhang, Jing Chen, Jie Zhou, Wei-Qing Liang, Pan Xu

**Affiliations:** 1 Development and Research Center of Official Silkworm Resources, Zhejiang Academy of Traditional Chinese Medicine, Hangzhou 310007, China; pjb0225@163.com (J.-B.P.); huyijuan604@163.com (Y.-J.H.); jian871211@sina.com (H.-J.Z.); Kenzei@163.com (J.Z.); jxlwq22@163.com (W.-Q.L.); 2 Key Laboratory of Research and Development of Chinese Medicine of Zhejiang Province, Hangzhou 310007, China; 3 College of Pharmacy, Hunan Chinese Medical University, Changsha 410208, China; xiabohou@163.com; 4 College of Life Science, Zhejiang Chinese Medical University, Hangzhou 310015, China; cj00123@zcmu.edu.cn

**Keywords:** *Rhizoma Atractylodes macrocephala* polysaccharides, ultrasonic-assisted enzymatic extraction, response surface methodology, desirability function approach, antioxidant activity, multi-optimization

## Abstract

*Rhizoma Atractylodes macrocephala* polysaccharides (RAMP) have been reported to have a variety of important biological activities. In this study, an ultrasonic-assisted enzymatic extraction (UAEE) was employed to obtain the highest extraction yield and strongest antioxidant activity of RAMP and optimized by a multi-response optimization process. A three-level four-factor Box-Behnken design (BBD) was performed as response surface methodology (RSM) with desirability function (DF) to attain the optimal extraction parameters. The DPPH scavenging percentage was used to represent the antioxidant ability of RAMP. The maximum D value (0.328), along with the maximum yield (59.92%) and DPPH scavenging percentage (13.28%) were achieved at 90.54 min, 57.99 °C, 1.95% cellulase and 225.29 W. These values were further validated and found to be in good agreement with the predicted values. Compared to the other extraction methods, both the yield and scavenging percentage of RAMP obtained by UAEE was favorable and the method appeared to be time-saving and of high efficiency. These results demostrated that UAEE is an appropriate and effective extraction technique. Moreover, RSM with DF approach has been proved to be adequate for the design and optimization of the extraction parameters for RAMP. This work has a wide range of implications for the design and operation of polysaccharide extraction processes.

## 1. Introduction

*Rhizoma Atractylodes macrocephala* (RAM) is the dried rhizome of *Atractylodes macrocephala* Koidz, which belong to the family Asteraceae (Compositae), found mainly distributed in China, Japan, and Korea [1]. RAM is widely used for the treatment of splenic asthenia, anorexia, oedema, excessive perspiration and abnormal fetal movement [2]. The rhizomes of *Atractylodes macrocephala* are rich in sesquiterpenes, polyacetylenes, phenylpropanoids, coumarins, glycosides and polysaccharides [1,3,4,5,6]. Recently, interest in *Rhizoma Atractylodes macrocephala* polysaccharides (RAMP) has been growing, because of the various bioactivities of RAMP, including immune response stimulating [7,8,9], aging defying [10], neuroprotective effects [11], hyperglycemic activity [12] and protective effects against liver ischemia reperfusion injury [13]. Most of the aforementioned activities are related with antioxidant properties and polysaccharides are widely considered to be a strong antioxidants.

However, little attention has been paid to the effective extraction of RAMP. Conventionally, polysaccharides are extracted by refluxing in hot water, which is often time-consuming, gives low yields of polysaccharides and even results in the loss of some of the pharmacological activity [14]. In recent years, various methods have been developed for the extraction of polysaccharides, such as ultrasonic-assisted extraction (UAE) and enzyme-assisted extraction (EAE). Among them, UAE is a rapid, energy efficient method that gives high extraction yields and has a minimal impact on the bioactivities [15]. Also, with the additional of specific enzymes such as cellulase and proteases, EAE can promote the release of intracellular contents by breaking the cell wall and lipid bodies [16]. Thus, it is considered as a mild, efficient and environmentally friendly extraction method, which has been used recently in the extraction of various kinds of compounds [17]. In this study, to achieve a high yield extraction and maximize the antioxidant ability of RAMP we combined the advantages of the UAE and EAE methods, in what is defined as ultrasonic-assisted enzymatic extraction (UAEE).

Response surface methodology (RSM) is an effective tool for optimizing experimental processes when many factors and interactions may affect the response variables [18]. Most of the work on RSM has been focused on the case where there is only one response of interest [15,17]. As a matter of fact, in many situations there are several response variables of interest or the relationship between the response variables and design variables is too complex to be efficiently estimated using traditional surface fitting approaches [19]. In this case, determination of the optimum settings on design variables would require simultaneous consideration of all the response [20], which is called a multi-response optimization (MRO).

Many creative methods have been developed for MRO, and the general purpose of these methods is to convert the MRO problem into a single aggregated objective function and then construct an efficient algorithm to find the optimum solution [21]. Presently, desirability function (DF) is a popular and established technique for the simultaneous determination of optimum settings of variables for multiple responses. It was first developed by Harrington and modified by Derringer and Suich [22,23]. It has been successfully used to optimize several multi-response cases, and its functions and advantages has been discussed in different articles [24,25,26].

However, the feasibility of using UAEE for RAMP and the multi-response optimization of the UAEE procedure by RSM mated with DF have not yet been explored in the literature. There is no research to emphasize the wide range of approaches that can be employed and the great impact on the polysaccharide extraction industries. Hence, this work aim to apply the UAEE method for the high yield and antioxidant activities of RAMP, and to find the optimal extraction condition using the RSM with DF approach in order to ensure rational utilization.

## 2. Results and Discussion

### 2.1. Single Factor Experimental Analysis of UAEE

All the factors that may affect the process must be carefully determined and examined. The experimental domain must be defined for each factor and also a control and measurement method must be established [27]. Thus, it is necessary to carry out single factor experiments to determine the experimental variables and interactions that have a significant influence on one or several responses. In this study, according to the priminary experiments, four major influencing factors, including extraction time, extraction temperature, cellulase concentration and ultrasonic power were selected for the next experiments.

#### 2.1.1. Effect of Extraction Time on the Yield of RAMP

**Figure 1:**
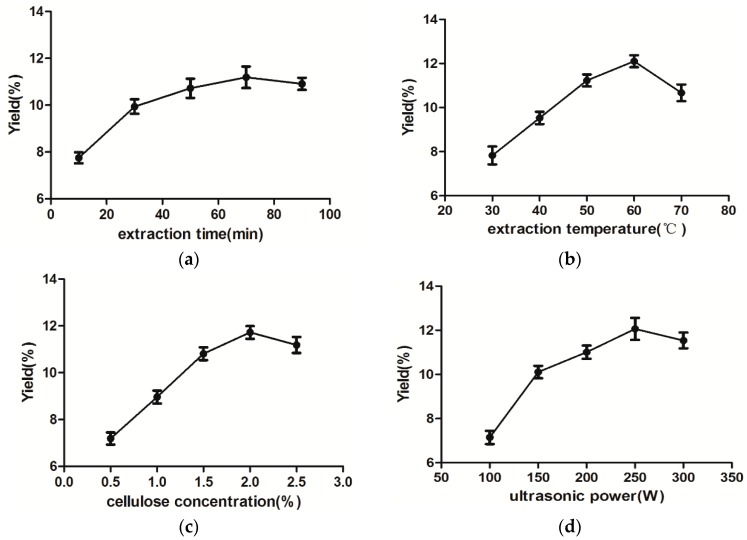
Effects of extraction time (**a**) extraction temperature (**b**) cellulase concentration (**c**) and ultrasonic power (**d**) on the extraction yield of RAMP (%).

Generally speaking, the longer the extraction time, the higher the yield of polysaccharides [28]. The effect of extraction time on extraction yield of RAMP is shown in Figure 1a. The yield of RAMP continued to increase gradually over the extraction time range of 10–70 min, until it reached its highest point at 70 min, which indicated that the polysaccharides were fully extracted as time goes by. When the extraction time reached 90 min, the increase in the yield of RAMP eased steadily. This phenomenon can be attributed to polysaccharide hydrolysis during the extended extraction [17].

#### 2.1.2. Effect of Extraction Temperature on the Yield of RAMP

The extraction temperature is an important factor influencing the extraction yield. As shown in Figure 1b, the yield of RAMP was significantly increased when the temperature was increased from 30 to 60 °C, and reached a peak value of 8.07%. Then further increase of extraction temperature resulted in a RAMP.extraction yield decease. One possible explanation is that the polysaccharides may be hydrolyzed at a high temperature [29], and the another reason is the activity of enzyme added to the solution were heavily influenced by the temperature, as enzyme activity is well known to be reduced when the temperature is too low or too high [30].

#### 2.1.3. Effect of Cellulase Concentration on the Yield of RAMP

Much research has shown that different cellulase concentrations will significantly affect the yield of polysaccharides [31,32]. As shown in Figure 1c the cellulase concentrationcurve indicated that the first increase of enzyme concentration lead to an obvious RAMP extraction yield increase till 2.0%, and then began to decrease slightly. This indicated that a 2.0% amount of enzyme was sufficient to obtain a high polysaccharide yield. Taking cellulase consumption and the yield of RAMP into account, the enzyme concentration of 2.0% was high enough for the UAEE.

#### 2.1.4. Effect of Ultrasonic Power on the Yield of RAMP

Ultrasonic power is a critical parameter in the UAEE method. Its effect on the extraction yield of RAMP is shown in Figure 1d. The yield increased as ultrasonic power increased from 100 W to 250 W, and decined when it went up to 300 W. The maximum extraction yield of RAMP was observed when the ultrasonic power was 250 W. A possible explaination is that the polysaccharides’ diffusion coefficient and the solubility of the polysaccharides in the extracting solvent increased with the enhanced ultrasonic power, and this eventually caused an incease in the amount of polysaccharide going out from the rhizome of *Atractylodes macrocephala* into solution [33]. After reaching the peak, the polysaccharide yield droped because of the loss during ultrasonicated post-processing [29]. To summarize the single-factor studies, the following conditions could used for the response surface methodology experiments: an extraction time of 10–90 min, an extraction temperature of 30–70 °C, a cellulase concentration of 0.5%–2.5% and a ultrasound power of 100–300 W.

### 2.2. Multi-Response Design and Analysis

#### 2.2.1. Statistical Analysis and Model Fitting

##### The yield of RAMP in the BBD experiments

As seen in Table 1, the results showed that the yield of polysaccharide (Y_1_) ranged from 7.22% to 13.10%.

**Table 1:** BBD matrix and response values for extraction yield and DPPH scavenging percentage of RAMP as well as D value.

Run	X_1_	X_2_	X_3_	X_4_	Y_1_^a^ (%)	Y_2_^a^ (%)	D Value
1	10	50	0.5	200	8.60 ± 0.63	39.11 ± 3.07	0.167
2	50	70	1.5	100	10.37 ± 0.52	50.51 ± 2.42	0.243
3	50	50	1.5	200	12.98 ± 0.64	63.13 ± 2.56	0.337
4	50	50	1.5	200	12.93 ± 0.65	63.26 ± 3.16	0.336
5	90	50	1.5	300	12.18 ± 0.62	54.16 ± 3.79	0.287
6	50	50	1.5	200	13.10 ± 0.59	63.87 ± 3.27	0.342
7	50	70	1.5	300	12.54 ± 0.60	57.74 ± 2.46	0.308
8	50	70	0.5	200	10.37 ± 0.57	46.60 ± 2.03	0.227
9	10	50	1.5	300	11.12 ± 0.70	48.70 ± 1.94	0.247
10	90	70	1.5	200	12.15 ± 0.50	56.10 ± 2.58	0.295
11	10	70	1.5	200	10.96 ± 0.56	48.23 ± 2.95	0.243
12	90	30	1.5	200	9.62 ± 0.71	46.13 ± 2.69	0.213
13	50	30	1.5	300	10.35 ± 0.63	51.13 ± 2.71	0.245
14	50	30	1.5	100	7.54 ± 0.55	42.29 ± 2.10	0.162
15	50	50	1.5	200	12.99 ± 0.72	63.88 ± 2.08	0.340
16	50	50	1.5	200	13.06 ± 0.70	63.51 ± 2.04	0.339
17	10	50	2.5	200	10.84 ± 0.59	49.07 ± 3.00	0.244
18	10	50	1.5	100	8.21 ± 0.74	41.75 ± 2.99	0.172
19	50	30	2.5	200	10.67 ± 0.58	49.64 ± 3.20	0.244
20	50	50	2.5	300	12.75 ± 0.50	55.66 ± 2.71	0.302
21	90	50	2.5	200	12.91 ± 0.72	55.63 ± 3.89	0.305
22	50	30	0.5	200	7.22 ± 0.53	42.47 ± 3.26	0.157
23	50	50	0.5	100	7.45 ± 0.50	39.72 ± 2.23	0.151
24	50	50	2.5	100	10.83 ± 0.51	51.88 ± 2.91	0.256
25	90	50	1.5	100	9.95 ± 0.70	48.11 ± 2.58	0.226
26	50	50	0.5	300	10.43 ± 0.45	50.52 ± 3.23	0.244
27	90	50	0.5	200	9.11 ± 0.67	45.91 ± 3.40	0.203
28	50	70	2.5	200	12.51 ± 0.50	59.84 ± 2.66	0.316
29	10	30	1.5	200	8.29 ± 0.80	43.83 ± 2.65	0.181

^a^ each value is the mean of triplicate measurements.

Multiple regression analysis was performed to build a mathematical model to find the optimum conditions that maximize the extraction yield of RAMP and study the relationship of the response variable and test variable. The second-order polynomial equation was given by the following expression:

(1) Y_1_ = 13.01 + 0.66X_1_ + 1.27X_2_ + 1.44X_3_ + 1.25X_4_ − 0.035X_1_X_2_ + 0.39X_1_X_3_ − 0.17X_1_X_4_ − 0.33X_2_X_3_ − 0.16X_2_X_4_ − 0.27X_3_X_4_ − 1.30X_1_^2^ − 1.47X_2_^2^ − 1.34X_3_^2^ − 1.33X_4_^2^

The analysis of variance (ANOVA), goodness-of-fit and the adequacy of the regression model were summarized in Table 2. The high model *F*-value and the low *P*-value indicates the level of confidence of the selected model [20]. The model *F*-value of 1337.56 and the associated lower *p*-value (*p* < 0.0001) implied the model was highly statistically significant. There was only a 0.01% chance that a “Model *F*-value” this large could occur due to noise. The value of R^2^ reflects the proportion of variation in the response attributed to the model rather than to random error [34]. The model has shown a good fit with the high R^2^ value and adjusted determination coefficient (R^2^_adj_) of 0.9993 and 0.9985, respectively. This confirmed that only 0.07% of the total variation is not explained and the experimental data were well fitted by the model. What’s more, the coefficient of the variation (C.V.) refer to the ratio of the standard error (SD) of estimated data to the mean value of the observed response [20]. In this model, the C.V. of 0.68% indicates that the simulation can be considered as resonably reproducible, which also means the precision and the experimental values of the model were of reliability. Table 2 also reveals that the interaction of extraction time and extraction temperature was insignificant (*p* > 0.05), while all other model terms were highly significant (*p* < 0.01). The results indicated that the linear coefficients (X_1_, X_2_, X_3_, X_4_), quadratic term coefficients (X_1_^2^, X_2_^2^, X_3_^2^, X_4_^2^) and cross product coefficients (X_1_·X_3_, X_1_·X_4_, X_2_·X_3_, X_2_·X_4_, X_3_·X_4_) were all significantly correlated with the RAMP extraction yield (*p* < 0.01).

**Table 2:** ANOVA for dependent variable: the yield of RAMP.

Source	Sum of Squares	df	Mean Square	F	Sig. (Prob > F)
Corrected model	100.64	14	7.19	1337.56	<0.0001
X_1_	5.20	1	5.20	967.75	<0.0001
X_2_	19.28	1	19.28	3587.29	<0.0001
X_3_	25.03	1	25.03	4656.98	<0.0001
X_4_	18.80	1	18.80	3498.22	<0.0001
X_1_X_2_	<0.0001	1	<0.0001	0.91	0.3558
X_1_X_3_	0.61	1	0.61	113.21	<0.0001
X_1_X_4_	0.12	1	0.12	21.51	0.0004
X_2_X_3_	0.43	1	0.43	79.83	<0.0001
X_2_X_4_	0.10	1	0.10	19.05	0.0006
X_3_X_4_	0.28	1	0.28	52.27	<0.0001
X_1_^2^	11.03	1	11.03	2052.10	<0.0001
X_2_^2^	14.07	1	14.07	2617.62	<0.0001
X_3_^2^	11.56	1	11.56	2151.64	<0.0001
X_4_^2^	11.50	1	11.50	2139.57	<0.0001
Residual	0.075	14	<0.0001		
Lack of fit	0.057	10	<0.0001	1.25	0.4493
Pure error	0.018	4	<0.0001		
Cor total	100.71	28			
R^2^	0.9993	SD	0.60		
R^2^_adj_	0.9985	C.V.%	0.68		
Adeq precision	110.900				

**Figure 2:**
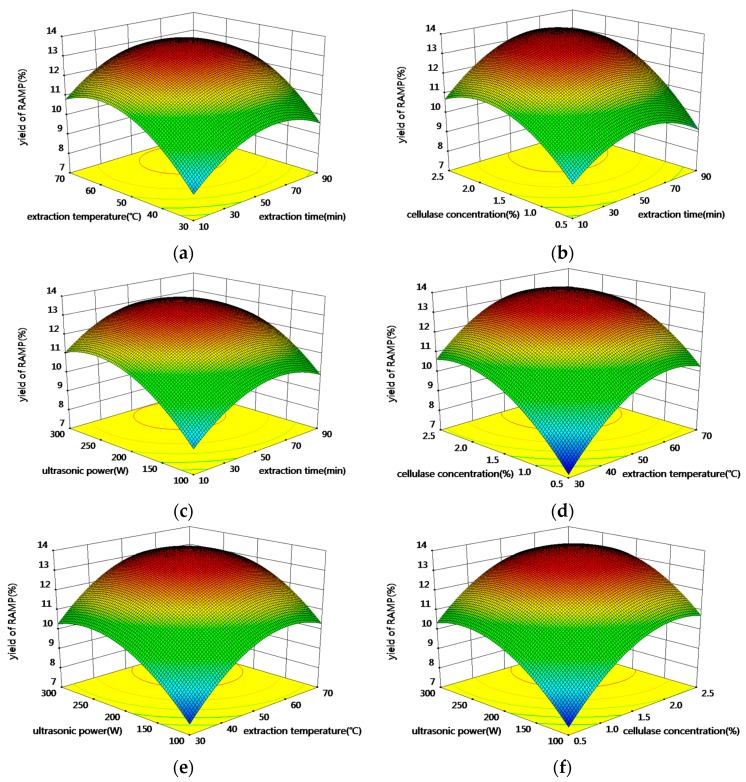
Response surface (3D) showing the effect of extraction parameters on extraction yield of RAMP. (**a**) extraction time and temperature; (**b**) extraction time and cellulase concentration; (**c**) extraction time and ultrasonic power; (**d**) extraction temperature and cellulase concentration; (**e**) extraction temperature and ultrasonic power; (**f**) cellulase concentration and ultrasonic power.

Response surfaces were plotted by the Design Expert software to explain the interactions of the variables for the maximum response. The corresponding three-dimensional response surfaces are shown in Figure 2. Each figure shows the effects of two factors at a time on the polysaccharide yield while all other factors were kept at zero level. Figure 2a illustrates the polysaccharide yield in response to extraction time and extraction temperature at a fixed ultrasound power of 200 W and a fixed celluose cncentration of 1.5%. The yield was very low at low extraction time and temperature, and increased as extraction time and extraction temperature increased until a peak value was reached. Further increasing the extraction time and temperature led to a decreased polysaccharide yield. Similar trends were observed for the effects of extraction time and cellulase concentration (Figure 2b), of extraction time and ultrasonic power (Figure 2c), of cellulase concentration and extraction temperature (Figure 2d), of ultrasonic power and extraction temperature (Figure 2e) and of ultrasonic power and cellulase concentration (Figure 2f). According to the model, the maximum yield of RAMP was 13.93% and the corresponding variables values were obtained after an extraction time of 61.91 min, at an extraction temperature of 56.99 °C, using a cellulase concentration of 2.00% and 237.96 W of ultrasonic power.

##### The DPPH Scavenging Percentage of RAMP in the BBD Experiments

As seen in Table 1, the DPPH scavenging percentage of RAMP (Y_2_) ranged from 39.11% to 63.88%. The data were analyzed by multiple regression analysis to get the following second-order polynomial equation:

(2) Y_2_ = 63.53 + 2.95X_1_ + 3.63X_2_ + 4.78X_3_ + 3.64X_4_ + 1.39X_1_X_2_ − 0.06X_1_X_3_ − 0.23X_1_X_4_ + 1.52X_2_X_3_ − 0.40X_2_X_4_ − 1.76X_3_X_4_ − 8.62X_1_^2^ − 6.40X_2_^2^ − 7.46X_3_^2^ − 6.69X_4_^2^

ANOVA results of the quadratic model presented in Table 3 show a high R^2^ of 0.9973 and a low C.V. value of 1.08%, demonstrating that the model can adequately describe the response surface of DPPH scavenging percentage. The high model *F*-value (376.23) and low *p*-value (*p* < 0.0001) suggested the results were highly statistically significant and had a good fit of the model. It can be seen from Table 3 that all the linear coefficients (X_1_, X_2_, X_3_, X_4_), quadratic term coefficients (X_1_^2^, X_2_^2^, X_3_^2^, X_4_^2^) and cross product coefficients (X_1_·X_2_, X_2_·X_3_, X_3_·X_4_) were significant model terms, with very small *p* value (*p* < 0.001). Moreover, the coefficients of X_1_·X_3_, X_1_·X_4_, X_2_·X_4_ were found non-significant (*p* > 0.05).

**Table 3:** ANOVA for the dependent variable: the DPPH scavenging percentage of RAMP.

Source	Sum of Squares	df	Mean Square	*F*	Sig. (Prob > *F*)
Corrected model	1629.04	14	116.36	376.23	<0.0001
X_1_	104.14	1	104.14	336.70	<0.0001
X_2_	157.91	1	157.91	510.55	<0.0001
X_3_	274.47	1	274.47	887.43	<0.0001
X_4_	158.78	1	158.78	513.37	<0.0001
X_1_X_2_	7.76	1	7.76	25.08	0.0002
X_1_X_3_	0.014	1	0.014	0.047	0.8323
X_1_X_4_	0.20	1	0.20	0.65	0.4320
X_2_X_3_	9.21	1	9.21	29.78	<0.0001
X_2_X_4_	0.65	1	0.65	2.10	0.1698
X_3_X_4_	12.32	1	12.32	39.83	<0.0001
X_1_^2^	482.07	1	482.07	1558.67	<0.0001
X_2_^2^	265.55	1	265.55	858.60	<0.0001
X_3_^2^	360.58	1	360.58	1165.86	<0.0001
X_4_^2^	290.38	1	290.38	938.89	<0.0001
Residual	4.33	14	0.31		
Lack of fit	3.86	10	0.39	3.27	0.1321
Pure error	0.47	4	0.12		
Cor total	1633.37	28			
R^2^	0.9973	SD	0.56		
R^2^_adj_	0.9947	C.V.%	1.08		
Adeq precision	60.809				

**Figure 3:**
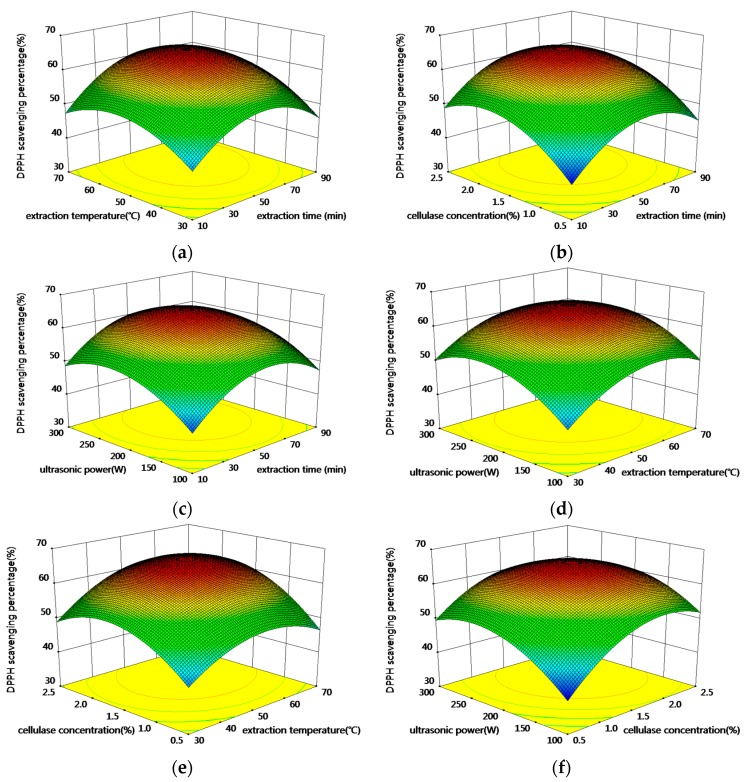
Response surface (3D) showing the effect of extraction parameters on DPPH scavenging percentage of RAMP. (**a**) extraction time and temperature; (**b**) extraction time and cellulase concentration; (**c**) extraction time and ultrasonic power; (**d**) extraction temperature and ultrasonic power; (**e**) extraction temperature and cellulase concentration; (**f**) cellulase concentration and ultrasonic power.

Figure 3a shows the DPPH scavenging percentage with varying extraction time and extraction temperature. From the figure, it can be seen that DPPH scavenging percentage increased as the extraction time and extraction temperature increased during the initial stage and then decreased slightly. Similar trends were observed for the other interactions of the model (Figure 3b–f). According to the model, the maximum DPPH scavenging percentage of 61.49% can be obtained after an extraction time of 85.53 min, at an extraction temperature of 58.29 °C, with a cellulase concentration of 1.84% and 220.05 W of ultrasonic power.

#### 2.2.2. Optimization Analysis of the UAEE Procedure

According to the previous study, the maximum value of RAMP yield by HWE was 3.13% [35]. We take the HWE maximum yield as our minimum polysaccharide yield in this study. The maximum value of the polysaccharide yield was specified as 50%. Besides, the maximum theoretical value of the DPPH scavenging percentage of RAMP is 100%, and the minimum DPPH scavenging percentage was specified as 20%. Thus, a one-sided transform of polysaccharides yield (*d*_1_) and DPPH scavenging percentage (*d*_2_) is obtained as follows:

(3) $$d_{1}=\left\{ \begin{array}{ll} 0 & Y_{i}\leq 3.13\\ \left[\frac{Y_{i}-3.13}{50-3.13}\right] & 3.13<Y_{i}<50\\ 1 & Y_{i}\geq 50 \end{array}\right.$$

(4) $$d_{2}=\left\{ \begin{array}{ll} 0 & Y_{i}\leq 20\\ \left[\frac{Y_{i}-20}{100-20}\right] & 20<Y_{i}<100\\ 1 & Y_{i}\geq 100 \end{array}\right.$$

The overall desirability *D* is calculated as:

(5) $$D=\sqrt{d_{1}d_{2}}$$

By using *D* as the new response, the optimum values of selected variables can be obtained through regression analysis. In this study, the optimal conditions for highest *D* (with a *D* value of 0.328) were: extraction time of 90.54 min, extraction temperature of 57.99 °C, cellulase concentration of 1.95% and 225.29 W of ultrasonic power. The corresponding maximum polysaccharide yield and DPPH scavenging percentage were 13.28% and 59.92%, respectively.

#### 2.2.3. Verification of the Predictive Model

To confirm the suitability of the model equation, three confirmation experiments were conducted under the optimized conditions. Considering the operability in actual production, the optimal conditions were slightly modified as follows: extraction time 90 min, extraction temperature 58 °C, cellulase concentration 1.95% and ultrasonic power 225 W. Under these conditions, the experimental yield of RAMP was 13.18% ± 0.56% (*n* = 3) and the DPPH scavenging percentage was 60.19% ± 2.99% (*n* = 3), which matched well with the predicted values of 13.28% and 59.92%, respectively. This confirmed that the model was adequate for optimization of the UAEE process. As a result, RSM coupled with DF approach was considered to be an accurate and decisive tool for predicting the maximum extraction yield and highest antioxidant activity of RAMP using the UAEE technique.

### 2.3. Comparison with Other Extraction Processes

**Figure 4:**
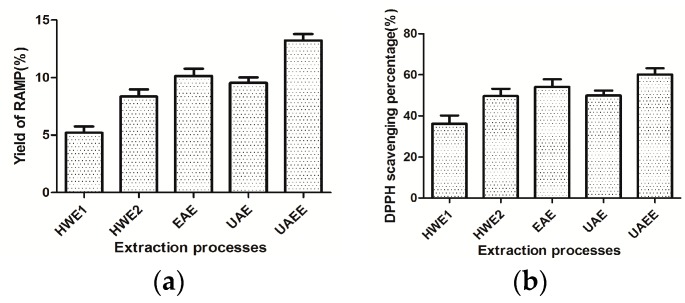
The yield (**a**) and DPPH scavenging percentage; (**b**) of RAMP of different extraction processes.

The results of our comparision with other extraction methods are illustrated in Figure 4. As shown in Figure 4a, the yield of RAM obtained by UAE and EAE was lower than that of UAEE for the same ratio of material and water, extraction time, temperature and pH. In addition, the yield of RAMP obtained by UAEE under the optimal conditions in this study was much higher and obtained faster than with the traditional hot water treatment, which obtained a yield of 5.21% under the conditons of 90 min extraction time and 58 °C temperature. When the extraction time was extended to 180 min and extraction temperature was went up to 80 °C, the RAMP yield by HWE rose to 8.35%, but was still lower than that of UAEE (13.18%). Consequently, UAEE has some obvious time-saving and high efficiency advantages when compared with other extraction processes. Figure 4b shows the DPPH sacvenging percentage among all the extraction processes, which obviously display a similar trend as the yield of RAMP. Thus, it was confirmed that UAEE should be an appropriate and effective extraction technique for obtaining high yields and maximum antioxidant activity of RAMP. Also, the costs of technique is so low that it has a very good industrial application prospects.

### 2.4. Preliminary Characterization of RAMP

**Figure 5:**
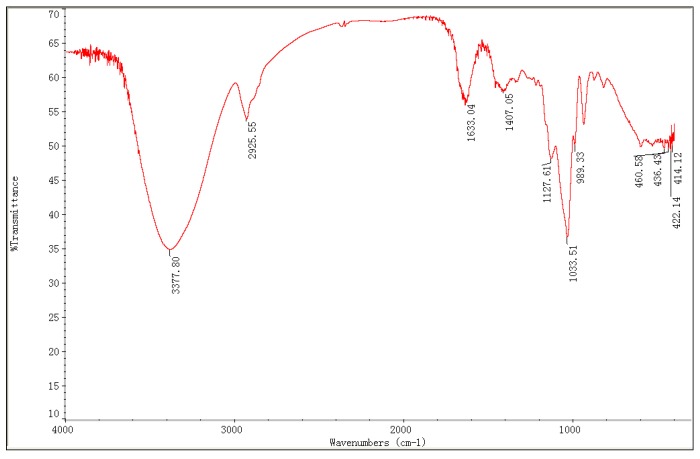
The FT-IR spectrum of RAMP.

The total neutral carbohydrate content of RAMP obtained by UAEE under the optimal conditions was estimated at 76.34% by the phenol-sulfuric acid method. RAMP had a negative response to the Bradford method. No absorption was observed at 280 nm in the UV spectrum, which also indicates the absence of protein. Its uronic acid content was under the limit of detection. The FT-IR spectrum of RAMP is shown in Figure 5. A strong broad stretching peak at approximately 3377.80 cm^−1^ for hydroxyl group and a weak band at approximately 2925.55 cm^−1^ for the C–H stretching vibration were observed; these two absorption bands are typical characteristic absorptions of polysaccharides [36]. The relatively weak absorption peaks at 1633.04 cm^−1^ and 1407.05 cm^−1^ also suggest the characteristic IR absorptions of polysaccharides [37]. The strong band in the region of 1000–1200 cm^−1^ was attributed to the C–O–C and C–OH bands in the IR spectrum, the peak of 1033.51 cm^−1^ suggests that the characteristic sugar moieties were of pyranose configuration [36]. Furthermore, no absorption peak was observed at 1740 cm^−1^, which indicates the absence of uronic acids in the polysaccharide structure [37]. This result further suggest that RAMP is a neutral polysaccharide.

## 3. Experimental Section

### 3.1. Materials

The rhizoma of *Atractylodes macrocephala* was purchased from the herb market of Pan’an Country, Zhejiang Province, China. It was authenticated by Professor Jinbao Pu (Zhejiang Academy of Traditional Chinese Medicine).

### 3.2. Chemical and Apparatus

The cellulase from *Trichoderma viride* (10,000 U/mg, lot#: C10008391)) was obtained from Shanghai Macklin Biochemical Co., Ltd. (Shanghai, China). d-Glucose and d-galacturonic acid (110833–200503, 111646–200301) were supplied by the National Institutes for Food and Drug Control (Beijing, China). Albumin (Bovine Serum) (10021008) was purchased from Shanghai Shenhang Biological Technology Co., Ltd. (Shanghai, China). 2,2-Diphenyl-1-picrylhydrazyl (DPPH; 100100723) was purchased from Sigma-Aldrich Chemical Company (St. Louis, MO, USA). All solvents and chemicals were at least of analytical grade and obtained from Sinopharm Chemical Reagent Co., Ltd. (Shanghai, China).

All UAEE experiments were carried out in an ultrasonic device (JK-300DB, Jiangsu, China) with a usable capacity of 10 L working at a frequency of 40 kHz and an ultrasound power of 100 W to 300 W. A centrifuge (Eppendorf 5415D, Hamburg, Germany) was used for the separation of the extract from the matrix residue. A rotary evaporator (Buchi R-210, Flawil, Switzerland) was used for the concentration of extracts. A UV-visible spectrophotometer (Varian Cary-100, Palo Alto, CA, USA) was used for the analysis of the carbohydrate content, protein content and uronic acid content of the RAMP. A microplate spectrophotometer (Biotek Powerwave XS, Winooski, VT, USA) was used for the analysis of DPPH free radical scavenging activity and a FT-IR spectrometer (Thermo Fisher Nicolet iS10, Waltham, MA, USA) was used for FT-IR spectra analysis of RAMP.

### 3.3. Methods

#### 3.3.1. UAEE Procedure

The herb material was reduced to a coarse powder with the help of a suitable grinder and passed through a 100 mesh screen. About 200 g of the dry rhizoma powder was doubly extracted with petroleum ether (boiling point: 60–90 °C) at 90 °C for 3 h each time and then pretreated in a Soxhlet extractor with 80% ethanol twice to remove lipids, some colored materials, monosaccharides and oligosaccharides [38]. The residue was then filtered and vacuum dried at 60 °C for 12 h for the following UAEE and conventional extraction.

Pretreated powder (1 g) was doubly extracted with cellulase solution (30 mL, using NaAc-HAc buffer system as our extracting solvent to adjust the pH at 4.6) at the given concentration (ratio of water to material 30 mL/g). The extraction time, extraction temperature, cellulase concentration and ultrasonic power was set according to the experimental design. After removing the matrix residue by centrifuge, the extraction solution was incorporated and concentrated to one-fifth of the initial volume using a rotary evaporator at 65 °C under reduces pressure. The concentrate was precipitated with the addition of anhydrous ethanol to a final concentration of 80% (*v*/*v*) and kept at 4 °C for 12 h. The precipitate was collected and deproteinated by the Sevage reagent (1-butanol/chloroform, *v*/*v* = 1:4) [39]. After mixing the water solution of polysaccharides and Sevage reagent together (*v*/*v* = 5:1), vibrated for 15 min and placed for 2 h to separating the denatured protein. The process was repeated three times and the product obtained then dried to get RAMP.

#### 3.3.2. Conventional Procedure

Hot water extraction (HWE), ultrasonic-assisted extraction (UAE) and enzyme-assisted extraction (EAE) of RAMP were also done to serve as the referance extraction methods. The specific conditions of the different extraction processes are listed in Table 4. All the extracts was obtained and treated according to the procedure mentioned in Section 3.3.1, and both of the yield and DPPH scavenging percentage were determinated.

**Table 4:** The differrent extraction process of RAMP.

Extraction Processes	Extraction Conditions				
	A	B	C	D	E
HWE1	-	-	90	58	7.0
HWE2	-	-	180	80	7.0
EAE	1.95	-	90	58	4.6
UAE	-	225	90	58	4.6
UAEE	1.95	225	90	58	4.6

A: cellulase concentration (%), B: ultrasonic power (W), C: extraction time (min), D: extraction temperature (°C), E: pH.

#### 3.3.3. Determination of the Yield of RAMP

The content of RAMP was measured by the phenol-sulfuric acid method [40] using d-glucose as a standard. The yield (%) of RAMP was then calculated as follows:

(6) $${\text{Y}}_{1}(\%)=\frac{\text{C}}{\text{W}}\times 100\%$$

where Y_1_ is the yield of RAMP, C is the weight of polysaccharides and W is the weight of raw material.

#### 3.3.4. Determination of Antioxidant Acitivity of RAMP

The antioxidant activity was studied through the evaluation of the free radical-scavenging effect on the DPPH radical, which was measured by the procedure described previously [41] with slight modification. Briefly, ascorbic acid (Vc) was used as the control, and 30 μL of RAMP solutions (0.1% in water) or control were mixed with 170 μL of the ethanolic DPPH (0.025 g/L). The mixture was shaken vigorously and incubated at 25 °C in the dark for 30 min. Absorption of the samples was measured on the microplate spectrophotometer at 517 nm. The scavenging percentage was calculated as follows:

(7) $${\text{Y}}_{2}(\%)=\frac{{\text{A}}_{c}-({\text{A}}_{i}-{\text{A}}_{j})}{{\text{A}}_{c}}\times 100\%$$

where *Y*_2_ is the scavenging percentage of RAMP, A*_c_* is the absorbance of DPPH solution without sample, A*_i_* is the absorbance of the sample mixed with DPPH solution and A*_j_* is the absorbance of the sample without DPPH solution.

#### 3.3.5. Experimental Design

##### Single Factor Experimental Design

The single factor experiment was performed in a designed conditions. During the optimization of experimental factors, one factor was changed while the other factors kept constant in each experiment. In this study, four factors including extraction time, extraction temperature, cellulase concentration and ultrasonic power were selected for the single factor experiments. In detail, the extraction time range was from 10 min to 90 min, the extraction temperature ranged from 30 °C to 70 °C, the cellulase concentration ranged from 0.5% to 2.5% (*w*/*v*) and the ultrasonic power range was from 100 W to 300 W. The effect of each factor was evaluated by determining the extraction yield of RAMP. All the experiments were repeated three times.

##### Box-Behnken Design

On the basis of the single factor experiments, a Box-Behnken design (BBD) with four factors and three levels was used for the further optimization of the UAEE conditions. Four independent variables including extraction time, extraction temperature, cellulase concentration and ultrasonic power were designated as X_1_, X_2_, X_3_ and X_4_, respectively. The ranges of values were based on the results of the preliminary experiments, as shown in Table 5. The response variables were the extraction yield of RAMP (Y_1_) and the DPPH scavenging percentage (Y_2_). As shown in Table 1, there were a total 29 runs based on the BBD with five center points performed in random order with triplicates in each run.

**Table 5:** Independent variables and their levels in Box-Behnken design

Independent variables	Symbol	Level		
		−1	0	1
Extraction time (min)	X_1_	10	50	90
Extraction temperature (°C)	X_2_	30	50	70
Cellulase concentration (%)	X_3_	0.5	1.5	2.5
Ultrasonic power (W)	X_4_	100	200	300

#### 3.3.6. Statistical Analysis and Optimization

The parameters of the response equation and analysis of variance (ANOVA) were performed by Design Expert Software (Version 8.0.6). A second order polynomial model used to fit the response to the independent variables is shown below:

(8) $$Y=\beta_{0}+\sum_{i=1}^{3}\beta_{i}X_{i}+\sum_{i=1}^{3}\beta_{ii}X_{i}^{2}+\sum \sum_{i<j}^{3}\beta_{ij}X_{i}X_{j}$$

where *Y* is the response, β_0_ is the intercept parameter and β*_i_*_,_ β*_ii_*_,_ β*_ij_* are the coefficients of the parameters for linear, squared and interaction effects, respectively.

The statistical significance for each term in the polynomial was evaluated by computing the *F*-value at a probability *p* of 0.05. The regression coefficients were then used to make statistical calculations and generate contour maps from the regression models. However, for multi-response, a desirability function approach can be used to transformed several response variables into a desirabitity function, which can be optimized by univariate techniques. A modified desirability approach, proposed by Derringer and Suich [22] is defined as:

(9) $$D={\left(d_{1}^{w_{1}}d_{2}^{w_{2}}d_{3}^{w_{3}}d_{4}^{w_{4}}d_{n}^{w_{n}}\right)}^{1/\sum w_{i}}$$

where *w_i_* is the relative weight of the *i*th response, *D* is the overall desirability , and *d_i_* is an individual response desiralibity. Then, the optimal setting is determined by the following, which is described previously [42,43].

(10) $$di=\left\{ \begin{array}{ll} 0 & Y_{i}\leq Y_{i-\min}\\ {\left[\frac{Y_{i}-Y_{i-\min}}{Y_{i-\max}-Y_{i-\min}}\right]}^{r} & Y_{i-\min}<Y_{i}<Y_{i-\max}\\ 1 & Y_{i}\geq Y_{i-\max} \end{array}\right.$$

where *Y_i_* is the response value, *Y_i-_*_min_ is the minimum acceptable value for response *i*, *Y_i_*_-max_ is the maximum acceptable value for response *i*, and *r* is a weight used to determine scale of desirability and equals 1 in this work.

#### 3.3.7. Preliminary Characterization of RAMP

The total neutral carbohydrate content was determinated by the phenol-sulfuric acid colorimetric method described in Section 3.3.3. The protein content was measured by Coomassie brilliant blue reaction [44] and the UV scanning spectrum at 280 nm. Uronic acid content was determinated by the *m*-hydroxydiphenyl method using d-galacturonic acid as standard [45]. The characteristic absorption of RAMP was identified by the FT-IR spectrum [36]. The RAMP powder was mixed with KBr powder, ground and pressed for FT-IR measurement in the wavenumber range from 400 to 4000 cm^−1^.

## 4. Conclusions

An UAEE process has been optimized for effective extraction of RAMP with high antioxidant activity. The maximum D value of 0.375, along with the maximum yield (13.73%) and scavenging percentage (62.18%) were achieved after an extraction time of 82.04 min, using an extraction temperature of 58.67 °C, a cellulase concentration of 1.99% and 230.67 W of ultrasonic power. These values were further validated by confirmatory experiments to see the efficacy of the model predictability and found to be in good agreement with the predicted values. Compared to other extraction methods, both the extraction yield and DPPH scavenging percentage of RAMP obtained by UAEE was favorable and the method appeared to be time-saving and of high efficiency. These results demostrated that UAEE is an appropriate and effective extraction technique for RAMP. Moreover, RSM with DF approach has been proved to be adequate for the design and optimization of the extraction parameters for RAMP. This works offer a wide range of implications for guiding the design and operation of polysaccharide extraction processes and may have a great impact on the polysaccharide extraction industries.
